# Book Reviews

**Published:** 1900-07

**Authors:** 


### Book Reviews.

THE PRINCIPLES OF TREATMENT AND THEIR APPLICATION TO THE PRACTICE OF MEDICINE. By J. MITCHELL BRUCE, M. D., F. R. C. P., Lecturer on the Practice of Medicine in the Charing Cross Hospital, London; Examiner in Medicine, Royal College of Physicians, London. Revised to conform with the U. S. Pharmacopœia by E. Q. THORNTON, M. D., Jefferson Medical College, Philadelphia. In one 8vo volume of 625 pages. Philadelphia: Lea Brothers & Co. 1900. [Price, $3.75.]

This work has been constructed on entirely new lines. Every student of medicine upon graduation deems himself a therapeutic master; he will be fortunate, however, if within a decade or two he does not become a therapeutic nihilist. Both of these extremes are to be deprecated. They might be avoided if books like this were written oftener and studied earlier.

The author asks a most pertinent question at the outset—one that occurs at the beginning of the treatment of every sick person. It is: What are the objects of treatment? Bruce, in writing this treatise, addresses himself to the answer of this question, and that he does so intelligently must be admitted by every one who reads the book. He does not commence with therapeutical laws—ready-made and fashioned to the case—but starts out to find them through the familiar facts of etiology, pathological anatomy and the clinical characters of disease. His chapter on the principles of treatment founded on etiology is a classical presentation of this elementary subject. In it he discusses the origin of disease and the natural resistance of the human body thereto, and then makes a searching look for etiological indications. In the chapter on principles of treatment founded on pathology, he pursues a similarly exhausted search for pathological indications. He makes the study exceedingly interesting by the novelty of his methods and the clothing of his ideas in attractive words.

Proceeding next to discuss the principles of treatment, founded on the clinical characters of disease, he presents a picture of transcendent interest described in elegant diction. One of the strong characteristics of the author is the forceful manner in which he discusses matters pertaining to convalescence—a subject that has heretofore received too little attention. An imprudence during the convalescent period often leads to the forfeiture of a life.

A short chapter is devoted to the principles of treatment founded on the personal factor in disease, and then comes one entitled, the proper relation of treatment to disease. In this chapter Bruce asserts that medicine can be justified by an appeal to nature as well as to sympathy. The therapeutic pessimist will gather small comfort from the teachings of this author. In speaking of the means of treatment, he gives a brief survey of the groups of remedial measures, but makes no attempt to discuss drugs as they fall under the department of materia medica and therapeutics, which Bruce has discussed in another volume. Everybody should read his chapter on the art of treatment He discusses this entirely apart from the science of treatment. One of his clear ideas drops suddenly upon the reader like a thunderbolt from a clear sky as follows:

"Personal experience and responsibility quickly undeceive the young practitioner. He finds that the theory of treatment and the practice of it as an art are two very different things. The practice of medicine and surgery is a real art: it means doing and using that which is best in the particular instance before us; not copying in a routine and mechanical fashion what has been done in other instances. Routine treatment is bound to fail more or less completely when put to the test of practice, even if based on sound, general principles."

The remaining chapters of the book are devoted to the consideration of illustrations of the principles of treatment. These are applied in a unique manner. At the end of each disease chosen to illustrate the principles taught by the author, an outline of practice is given, which is a summary of the whole proceeding from the commencement of the case to the end of convalescence. A well-prepared index brings to a close this admirable volume.

This is one of the recent works for which there is ample room and no apology need be made for its appearance at this time. It is one of the most helpful books to the general practitioner of medicine that has been published within a decade.

Progressive Medicine. Volume II., 1900. A Quarterly Digest of Advances. Discoveries and Improvements in the Medical and Surgical Sciences, Edited by Hobart Amory Hare, M. D., Professor of Therapeutics and Materia Medica in Jefferson Medical College of Philadelphia. Octavo, handsomely bound in cloth, 401 pages with 81 engravings. Issued quarterly. Philadelphia and New York: Lea Brothers & Co. [Price, $10 per year.]

This volume contains articles on surgery of the abdomen, including hernia; gynecology; diseases of the blood; diathetic and metobolic diseases; diseases of the glandular and lymphatic system, and upon ophthalmology.

The first named subject is presented by Dr. William B. Coley and occupies the first 132 pages of the book. In it he describes the newest phases of the surgery of the stomach, (the whole subject being measurably new) appendicitis, hernia, and abdominal tumors, added to which is a delineation of the use of the *x*-ray in the detection of abdominal calculi. The illustrations of this section are especially to be commended for their number, accuracy, and clearness.

The article on gynecology is written by Dr. John G. Clark and, as might be expected, is chiefly a résumé of the work of the Johns

Hopkins School and its coterie of admirers. On the topic "Kraurosis vulvæ," for example, we fail to discover any reference to the writings of Charles A. L. Reed, who was one of the first to describe the condition with accuracy; or to Howard W. Longyea's paper, which followed up the topic with later data. The subjects, however, are well presented and some of the illustrations are good.

The section on diseases of the blood and disorders of metabolism, is written by Dr. Alfred Stengel. It is needless to add that the whole subject is admirably and instructively presented. The most valuable literature of the entire field has been gleaned and the section is of great practical interest.

The section devoted to the consideration of ophthalmology is prepared by Dr. Edward Jackson. It is a carefully selected compendium of recent advances in diseases of the eye and is particularly adapted to the needs of the general practitioner of medicine.

The year's events in these several fields have been well compiled and the volume is a distinct addition to the library needs of busy physicians.

A POCKET MEDICAL DICTIONARY. Giving the pronunciation and definition of the principal words used in medicine and the collateral sciences, including very complete tables of clinical eponymic terms; of the arteries, muscles, nerves, bacteria, bacilli, micrococci, spirilla and thermometric scales; and a dose-list of drugs and their preparations in both the English and metric systems of weights and measures. By GEORGE M. GOULD, A. M., M. D. Fourth edition, revised and enlarged, containing 30,000 words. Philadelphia: P. Blakiston's Son & Co. 1900. [Price, $1.00.]

This excellent pocket dictionary has been constantly improved with each edition since it first appeared, until now it leaves little to be desired in the line of its particular sphere of usefulness. It is well printed in clear type, on good paper, and is bound in strong, black morocco, flexible covers. It first contained about 12,000 words and now defines some 30,000 medical terms, which amply equips the student or practitioner with a handy lexicon that is brought down to date in all essentials for the class-room, laboratory, or office desk. We commend it most cordially to everybody who wants such a condensation for ready reference; and who does not?

